# Sex disparity in the association between alcohol consumption and sarcopenia: a population-based study

**DOI:** 10.3389/fnut.2025.1536488

**Published:** 2025-02-07

**Authors:** Longbao Yang, Qiuju Ran, Yee Hui Yeo, Zhang Wen, Shuyue Tuo, Yong Li, Jia Yuan, Shejiao Dai, Jinhai Wang, Fanpu Ji, Xinxing Tantai

**Affiliations:** ^1^Department of Gastroenterology, The Second Affiliated Hospital of Xi'an Jiaotong University, Xi'an, China; ^2^Clinical Research Center for Gastrointestinal Diseases of Shaanxi Province, The Second Affiliated Hospital of Xi'an Jiaotong University, Xi'an, China; ^3^Karsh Division of Gastroenterology and Hepatology, Cedars-Sinai Medical Center, Los Angeles, CA, United States; ^4^Department of Infectious Diseases, The Second Affiliated Hospital of Xi'an Jiaotong University, Xi'an, China; ^5^National & Local Joint Engineering Research Center of Biodiagnosis and Biotherapy, The Second Affiliated Hospital of Xi'an Jiaotong University, Xi'an, China; ^6^Shaanxi Provincial Clinical Research Center for Hepatic & Splenic Diseases, The Second Affiliated Hospital of Xi'an Jiaotong University, Xi'an, China; ^7^Key Laboratory of Environment and Genes Related to Diseases, Xi'an Jiaotong University, Ministry of Education of China, Xi'an, China

**Keywords:** alcohol, sarcopenia, sex, NHANES, cross-sectional study

## Abstract

**Background:**

Previous studies have shown inconsistent findings regarding the association of alcohol consumption with sarcopenia. Therefore, this study comprehensively investigated the association of alcohol consumption with sarcopenia in a nationally representative sample of US adults.

**Methods:**

This population-based study included adults aged 18 years and older from the National Health and Nutrition Examination Survey (NHANES) III. Alcohol exposure was defined as daily alcohol intake, alcohol drinking history, number of drinking days per week, and frequency of binge drinking days per month. Weighted logistic regressions were used to determine associations.

**Results:**

Four cohorts were selected from the NHANES III: cohort 1 (*n* = 7,592), cohort 2 (*n* = 12,060), cohort 3 (*n* = 7,608), and cohort 4 (*n* = 7,649), corresponding to alcohol exposure categories of daily alcohol intake, drinking history, number of drinking days per week, and frequency of binge drinking days per month. In the full model, the risk of sarcopenia was significantly associated with mild (odds ratio [OR]: 1.65; 95% confidence interval [CI]: 1.08–2.51), moderate (OR: 2.04; 95% CI: 1.12–3.71), and heavy drinkers (OR: 2.42; 95% CI: 1.17–4.97) compared to nondrinkers. There was an association between the development of sarcopenia and current drinkers (OR: 1.69; 95% CI: 1.12–2.56) but not former drinkers (OR: 1.21; 95% CI: 0.88–1.66). Compared to nondrinkers, an increased risk of developing sarcopenia was observed in participants who consumed alcohol 2 days (OR: 2.36; 95% CI: 1.40–3.99) or > 2 days (OR: 1.84; 95% CI: 1.10–3.07) per week, and those who engaged in binge drinking for ≤1 day per month (OR: 1.68; 95% CI: 1.09–2.60) or > 1 day per month (OR: 2.10; 95% CI: 1.10–4.01). Sensitivity analyses based on different definitions of sarcopenia yielded similar results. Stratified analyses revealed that these associations were present in females but not males.

**Conclusion:**

Alcohol intake was associated with an increased risk of sarcopenia in all individuals, with this association being primarily observed in females rather than males.

## 1 Introduction

Sarcopenia is a skeletal muscle disorder characterized by the progressive and generalized depletion of muscle mass and/or function (1). It represents a broader syndrome distinct from muscular dystrophy, which is a clinically and genetically heterogeneous group of inherited diseases. In the 1980s, sarcopenia was initially characterized as a decline in lean body mass associated with age, affecting mobility, nutritional status, and independence (2). As a muscle disease, sarcopenia is rooted in adverse muscle changes that accumulate over a lifetime. It is prevalent among older adults but can also occur earlier in life (3). A recent meta-analysis revealed a prevalence of sarcopenia ranging from 8 to 36% in individuals under the age of 60 and from 10 to 27% in those aged 60 and older, with a notable sex difference (4). The prevalence of sarcopenia is higher in women than in men, not only in the general population but also in populations with cardiovascular diseases (5, 6). The risk factors for sarcopenia are also sex-specific (6). In addition to age-related primary sarcopenia, secondary sarcopenia is highly prevalent in patients with conditions such as bedridden status, kidney disease, liver disease, inflammatory disease, malignancies, and malnutrition (3). More notably, recent research has indicated a significant association between sarcopenia and various adverse outcomes such as falls, functional decline, frailty, cardiovascular disease, liver fibrosis, cancer, and mortality (1, 7–9), which imposes a considerable economic burden on the healthcare system (10).

Alcohol use, identified as one of the 10 leading risk factors for the global burden of disease, is a well-known contributor to disability and mortality in both men and women across all age groups, particularly when consumed excessively (11, 12). Despite previous global initiatives to reduce harmful alcohol use, global alcohol consumption has not decreased over the past three decades, and predictions anticipate an increase until at least 2030 (13). Alcohol remains one of the most widely consumed psychoactive substances worldwide, with 2.4 billion current drinkers (14). Alcohol consumption can cause various diseases such as liver disease, cancer, cardiovascular disease, and malnutrition/sarcopenia (15, 16).

The association between alcohol intake and sarcopenia remains a subject of debate. *In vitro* and *in vivo* studies have shown that excessive alcohol consumption increases the risk of sarcopenia via direct and indirect mechanisms that impact skeletal muscle protein metabolism (16). These mechanisms primarily involve alcohol-induced detrimental impacts, including gut microbiota dysbiosis, increased muscle autophagy, and heightened systemic inflammation and insulin resistance (16). However, this phenomenon has not been fully confirmed in clinical or epidemiological studies. Some studies suggested that alcohol intake significantly increased the risk of developing sarcopenia or muscle depletion (17–19). However, other studies indicated that this association was not statistically significant (20–22). Certain studies have even proposed that alcohol consumption significantly reduces the risk of sarcopenia (23, 24). A 2016 meta-analysis of 13 studies with individuals over the age of 65 found that alcohol intake served as a protective factor against the development of sarcopenia in males and the overall population (25). Subsequently, an updated meta-analysis of 19 observational studies published in 2022 reported that alcohol consumption was not significantly associated with the risk of sarcopenia (26). In light of the current research, there is a need for further clarification of the association between alcohol consumption and sarcopenia (1, 27). Therefore, we comprehensively assessed the association between risk of sarcopenia and alcohol drinking including alcohol consumption, drinking history, drinking frequency, and binge drinking frequency in a nationally representative sample of US adults. Additionally, women seem to have a greater vulnerability to the detrimental effects of ethanol compared to men, possibly due to their lower *χ*-alcohol dehydrogenase activity (28). Previous studies have shown that female drinkers are more prone to developing alcoholic liver disease, alcohol-related heart disease, and cancer at the same level of alcohol exposure as men (29–31). In light of this, the study also aimed to explore the impact of sex on the association between alcohol exposure and sarcopenia.

## 2 Methods

### 2.1 Study population

This population-based study was based on data obtained from the National Health and Nutrition Examination Survey (NHANES) III. The NHANES III, conducted by the National Center for Health Statistics (NCHS) from 1988 to 1994, is a study program using a stratified, multistage clustered design with the aims of assessing the health and nutritional status of noninstitutionalized civilians in the US (32). The study was approved by the institutional review board of the NCHS. After providing written informed consent, participants underwent personal interviews, physical examination, and laboratory tests. This study included participants aged 18 years and older at baseline with complete information on sarcopenia and alcohol exposure. All data used in the survey are publicly available online through the NHANES website.^1^

### 2.2 Assessment of alcohol drinking

Standardized questionnaires were used to extract information related to alcohol use status and patterns of consumption. Participants who responded “no” to having consumed at least 12 alcoholic drinks throughout their entire lifetime and in the past 12 months were categorized as “nondrinkers.” Those who responded “yes” to having consumed at least 12 alcoholic drinks in their entire lifetime but not in the past 12 months were categorized as “former drinkers.” Respondents who answered “yes” to having consumed at least 12 alcoholic drinks in the past year or in their entire lifetime and had consumed alcohol on at least 1 day in the past year were categorized as current drinkers (33–35). Participants provided information on the average number of drinking days and average number of drinks per drinking day over the past 12 months. The calculation of the average number of drinks per day was derived from these reported values (36). Alcohol drinking levels were categorized based on daily intake as mild (≤ 1 drink), moderate (1–2 drinks for women or 1–3 drinks for men), or heavy (> 2 drinks for women or > 3 drinks for men) (37). Alcohol drinking days per week were calculated based on the average number of drinking days in the past 12 months and further characterized as low (≤ 1 day), moderate (1–2 days), or high (> 2 days) frequency. Binge drinking days per month were calculated based on the question: “How many days did you have five or more drinks per day in the past 12 months?” and further classified as low (≤ 1 day) or high (> 1 day) frequency (38, 39).

### 2.3 Assessment of sarcopenia

In the NHANES III database, whole body bioimpedance analysis (BIA) was measured as the resistance at 50 kHz between the right wrist and ankle of a supine participant using the Valhalla 1990B Bio-Resistance Body Composition Analyzer (Valhalla Medical, San Diego, CA, United States). Skeletal muscle mass (SMM) was assessed using the formula developed by Janssen et al. (40), which has been validated through comparisons with skeletal muscle mass measured by magnetic resonance imaging (40). The formula is expressed as follows: SMM(kg) = height^2^ / BIA - resistance x 0.401 + (sex × 3.825) + (age × –0.071) + 5.102. In this formula, height is measured in centimeters, BIA resistance is documented in ohms, sex is coded as 1 for men and 0 for women, and age is recorded in years. This formula has been widely utilized for the assessment of sarcopenia in the NHANES III study (41, 42). The skeletal muscle index (SMI) was calculated as the absolute SMM (kg) divided by height (m^2^). Participants were defined as having sarcopenia if their SMI was more than two standard deviations below the sex-specific mean for young adults (18–39 years) in NHANES III, in accordance with the cutoff recommendations of the European Working Group on Sarcopenia in Older People (EWGSOP) 2 (3). The calculated SMI cutoff values for sarcopenia were <8.42 kg/m^2^ for males and <6.06 kg/m^2^ for females, which closely resembled the BIA-SMI cutoff values recommended by the EWGSOP guideline (<8.87 kg/m^2^ for males and <6.42 kg/m^2^ for females) (43). Most guidelines on sarcopenia recommend that the definition of sarcopenia consider both low SMI and low muscle strength or low physical performance (44, 45). NHANES III lacks data on muscle strength, but certain participants underwent a timed 8-foot walk test to assess physical performance. Low physical performance was defined as a gait speed <1.0 m/s (44, 45). As a sensitivity analysis, sarcopenia was alternatively defined as low SMI and low physical performance (44, 45).

### 2.4 Assessment of covariates

Household income was assessed using the ratio of family income to poverty, which is calculated by dividing the total family income in the previous year by the poverty threshold specific to the survey year. Participants who reported smoking fewer than 100 cigarettes over their lifetime were labeled as nonsmokers, whereas those who had smoked ≥100 cigarettes were labeled as smokers (46). Dietary interviews, involving a 24-h recall of intake, were conducted by trained interviewers to collect details on specific foods and quantities. Daily energy and protein intake were calculated based on the U.S. Department of Agriculture Survey Nutrient Database guidelines. Participants with daily energy intake (≥30 kcal/kg) and protein intake (≥1.0 g/kg) were considered to meet the requirements based on the nutritional guidelines for sarcopenia (47). Physical activity questionnaires were administered to all participants to collect information about the frequency of leisure activities (e.g., walking, dancing, and swimming) in the previous month. Using the Compendium of Physical Activities (48), the intensity of each activity was measured by metabolic equivalent (MET), with one MET defined as the energy expenditure at the resting metabolic rate. The intensity of the activity was further categorized as low (≤3), moderate (3–6), or vigorous (>6) based on their MET value (41, 42). Inactive participants were defined as those who engaged in no physical activity during their leisure time. Active participants were categorized as individuals who exercised at a moderate intensity with a frequency of ≥5 times per week, engaged in high-intensity exercise with a frequency of ≥3 times per week, or accumulated a total frequency of ≥5 times per week through a combination of moderate and high-intensity activities. Participants not meeting these criteria were classified as moderate (49). Serum 25(OH)D levels below 50 nmol/L were defined as vitamin D deficiency (50), and serum C-reactive protein (CRP) levels exceeding 0.3 mg/dL were considered indicative of high systemic inflammation (51). Hypertension was defined as blood pressure ≥ 140/90 mmHg, self-reported medical history, or current use of antihypertensive drugs (36). Diabetes mellitus was defined as meeting any of the following criteria: self-reported medical history of diabetes, self-reported use of insulin or oral hypoglycemic agents, fasting plasma glucose ≥126 mg/dL, 2-h plasma glucose ≥200 mg/dL, or hemoglobin A1C ≥ 6.5% (36). Hyperlipidemia was defined as self-reported use of lipid-lowering drugs, total cholesterol levels ≥200 mg/dL, or triglyceride levels ≥150 mg/dL (52). Chronic kidney disease was identified as estimated glomerular filtration rate < 60 mL/min/1.73 m^2^ (53). The diagnosis of cancer was defined by inquiring, “Has a doctor ever told you that you had any cancer?” Chronic lung disease was diagnosed by inquiring, “Has a doctor ever told you that you had asthma, chronic bronchitis, or emphysema?” Cardio-cerebrovascular disease was diagnosed by inquiring, “Has a doctor ever told you that you had a heart attack, congestive heart failure, or stroke?”

### 2.5 Statistical analyses

All statistical analyses followed the Centers for Disease Control and Prevention guidelines, with sampling weights, strata, and clusters considered due to the complex multistage stratified probability survey design used in the NHANES III (54). Continuous variables were presented as the median, while categorical variables were expressed as percentages. The generalized linear regressions, and χ^2^ tests were used to compare the differences between the sarcopenia and nonsarcopenia groups for continuous and categorical variables, respectively. Directed acyclic graphs (DAGs) were created to identify confounders of the association between alcohol consumption and sarcopenia, taking into consideration expert knowledge and current evidence from the literature (55). As shown in Supplementary Figure S1, we considered the following variables as adjusting covariates: age, sex, ethnicity, education level, and marital status.

To investigate the dose–response relationship between the number of drinks per day and risk of developing sarcopenia, restricted cubic spline regression was conducted with three knots in all participants and stratified by sex, adjusting for all covariates identified by the DAG. The independent association between daily alcohol consumption and sarcopenia was assessed by weighted logistic regression. Three models were constructed: Model 1, unadjusted; Model 2, adjusted for age and sex; and Model 3, the full model, adjusted for age, sex, ethnicity, education level, and marital status. We also conducted subgroup analyses according to age (grouped by median, ≤ 40/> 40), ethnicity (Non-Hispanic White, Non-Hispanic Black, Mexican-American, Others), education level (high school and higher/less than high school), married (yes/no), smoking (yes/no), physical activity level (inactive, moderate, active), number of comorbidities (≤ 1/> 1), household income level (< 1.3/≥ 1.3), body mass index (BMI) (< 25/≥ 25), energy or protein intake meet requirements (yes/no), vitamin D deficiency (yes/no), and CRP level (≤ 0.3/> 0.3). Subgroup interaction analyses were performed to assess the difference between groups.

The associations between alcohol intake and sarcopenia were further evaluated by daily alcohol consumption level (nondrinkers, mild, moderate, and heavy), alcohol drinking history (nondrinkers, former drinkers, and current drinkers), alcohol drinking days per week (continuous/categorical), and binge drinking days per month (continuous/categorical). Linear trend tests were performed with the categorical variables of interest defined on a continuous scale. A sensitivity analysis was conducted using an alternative definition of sarcopenia, which considered both SMI and gait speed. Another sensitivity analysis was performed based on the full model, with additional adjustments for daily protein intake, physical activity level, daily polyunsaturated fatty acid intake, daily zinc intake, vitamin D level, and CRP. Furthermore, all analyses were stratified by sex to evaluate potential differences between men and women in these associations. All statistical analyses were conducted using R (version 4.1.2; R Foundation for Statistical Computing, Vienna, Austria). Two-tailed *p* < 0.05 was considered statistically significant.

## 3 Results

**Figure 1:**
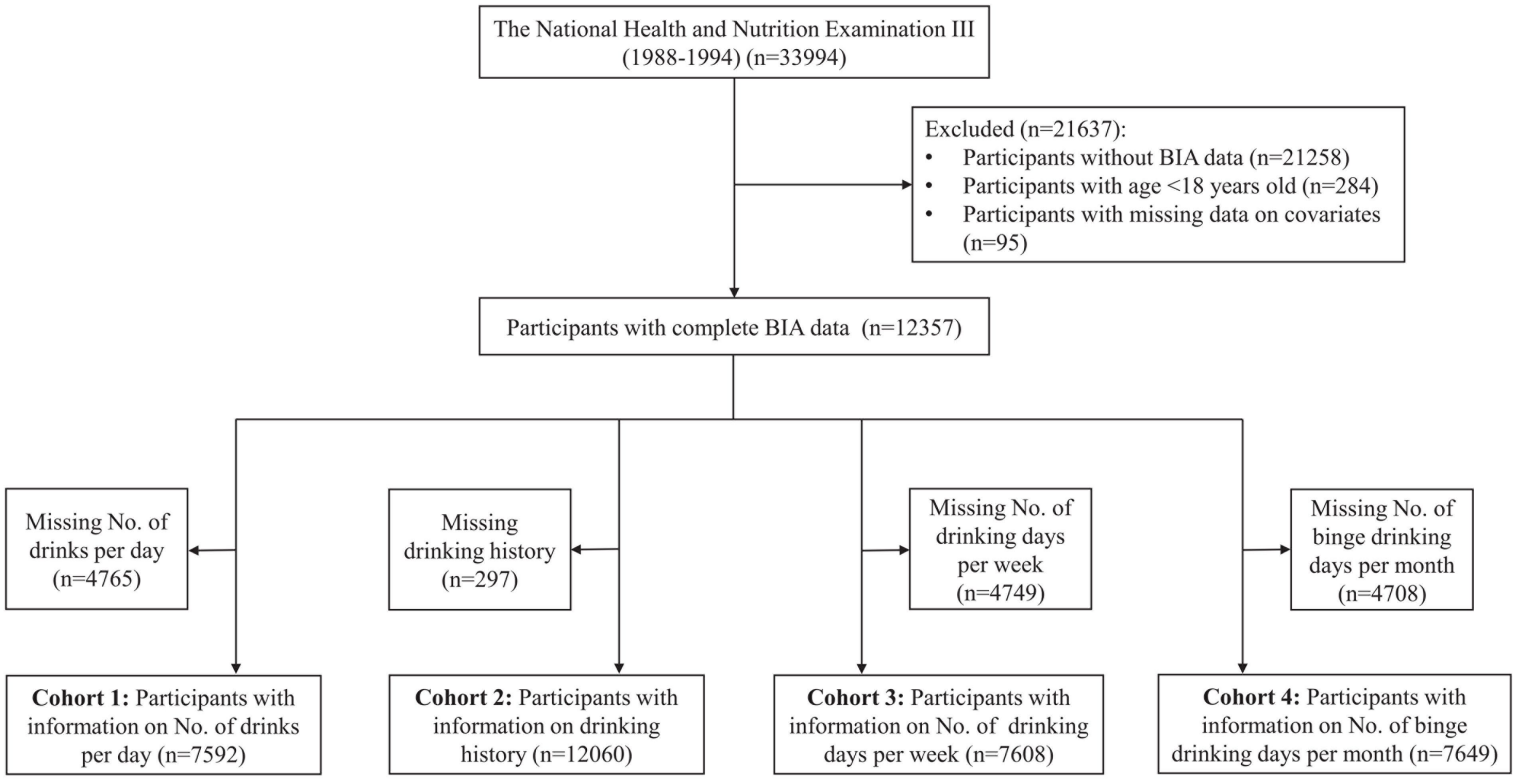
Flow chart of participants included in the study. BIA, bioimpedance analysis.

Alcohol exposure was assessed as daily alcohol intake, alcohol drinking history, number of drinking days per week, and binge drinking days per month, corresponding to four different cohorts: cohort 1 (*n* = 7,592), cohort 2 (*n* = 12,060), cohort 3 (*n* = 7,608), and cohort 4 (*n* = 7,649), respectively (Figure 1). The baseline characteristics of the study population (cohort 1) are shown in Table 1. Overall, the median age was 38 (28–52) years, and more than half of the participants were male (53.5%). The participants were mostly Non-Hispanic White, married, smokers, and had at least a high school education. The distribution of participants across inactive, moderate, and active levels was 12.1, 44.4, and 43.5%, respectively. The percentages of nondrinkers, mild drinkers, moderate drinkers, and heavy drinkers were 19.2, 57.8, 16.7, and 6.3%, respectively. Approximately 48.1% of participants had two or more comorbidities. The median number of days of alcohol consumption per year was 52, with a median alcohol consumption of two drinks on drinking days. The median BMI was 25 kg/m^2^, and the median SMI was 9.3 kg/m^2^. Participants with sarcopenia were more likely to be older, female, have a lower education level, lower BMI, lower levels of energy or protein intake, lower levels of vitamin D, lower levels of physical activity, higher number of comorbidities, and more days of alcohol consumption per year compared to those without sarcopenia. The baseline characteristics of the other cohorts are presented in Supplementary Tables S1–S3.

**Table 1:** Baseline characteristics of the study population (cohort 1) according to sarcopenia status.

Variable	Total (*n* = 7,592)	Nonsarcopeinia (*n* = 7,146)	Sarcopenia (*n* = 446)	*p* value
Estimate U.S population (*N*)	89,905,302	86,136,237	3,769,065	
Age, median (IQR), y	38 (28,52)	37 (28,50)	64.00 (50,75)	< 0.001
Sex, *n* (%)				< 0.001
Female	3,603 (46.5)	3,349 (45.7)	254 (64.9)	
Male	3,989 (53.5)	3,797 (54.3)	192 (35.1)	
Ethnicity, *n* (%)				0.070
Mexican-American	2047 (4.8)	1977 (4.9)	70 (2.2)	
Non-Hispanic Black	2030 (9.9)	1935 (9.9)	95 (9.0)	
Non-Hispanic White	3,202 (77.3)	2,937 (77.1)	265 (83.3)	
Others	313 (8.0)	297 (8.1)	16 (5.5)	
Married, *n* (%)				0.110
No	3,112 (37.3)	2,888 (37.1)	224 (42.7)	
Yes	4,480 (62.7)	4,258 (62.9)	222 (57.3)	
Education level, *n* (%)				< 0.001
High school or higher	6,036 (90.6)	5,748 (91.1)	288 (79.4)	
Less than high school	1,556 (9.4)	1,398 (8.9)	158 (20.6)	
Smoking, *n* (%)				0.140
No	3,912 (47.2)	3,696 (47.4)	216 (43.3)	
Yes	3,680 (52.8)	3,450 (52.6)	230 (56.7)	
BMI, median (IQR), kg/m^2^	25.0 (22.2,28.5)	25.1 (22.4,28.6)	21.5 (19.2,23.7)	< 0.001
Poverty income ratio, median (IQR)	3.0 (1.8,4.4)	3.0 (1.8,4.4)	2.9 (1.6,4.5)	0.500
Energy intake, median (IQR), kcal/d	2088 (1,536,2,858)	2,113 (1,558,288)	1,591 (1,200,211)	< 0.001
Protein intake, median (IQR), g/d	75.2 (54.0,103.7)	76.2 (54.4,104.8)	61.6 (43.9, 82.0)	< 0.001
Vitamin D levels, median (IQR), nmol/L	62.8 (46.8,80.3)	62.8 (47.0,80.5)	59.2 (42.4,77.4)	0.020
Total polyunsaturated fatty acids intake (g/d)	15.2 (9.2,24.3)	15.4 (9.4,24.6)	10.7 (6.8,17.5)	< 0.001
Zinc intake (mg/d)	10.5 (7.0,14.9)	10.6 (7.0,15.1)	8.4 (5.7,11.6)	< 0.001
CRP, median (IQR), mg/dL	0.2 (0.2,0.2)	0.2 (0.2,0.2)	0.2 (0.2,0.4)	0.060
Physical activity level, *n* (%)				< 0.001
Inactive	1,392 (12.1)	1,251 (11.6)	141 (23.5)	
Moderate	3,196 (44.4)	3,033 (44.5)	163 (41.6)	
Active	3,004 (43.5)	2,862 (43.9)	142 (34.9)	
Number of comorbidities, *n* (%)				< 0.001
0–1	3,693 (51.9)	3,596 (53.1)	97 (24.9)	
≥2	3,899 (48.1)	3,550 (46.9)	349 (75.1)	
No. of drinking days per year, median (IQR)	52 (24,156)	52 (24,156)	104 (48,260)	0.001
No. of drinks per day on drinking day, median (IQR)	2 (2,4)	2 (2,4)	2 (1,3)	< 0.001
No. of drinks per day, median (IQR)	0.3 (0.1,0.9)	0.3 (0.1,0.9)	0.3 (0.0,1.0)	0.170
Alcohol drinking levels, *n* (%)				< 0.01
Nondrinkers	2,144 (19.2)	1962 (18.8)	182 (28.5)	
Mild	3,811 (57.8)	3,626 (58.2)	185 (50.5)	
Moderate	1,189 (16.7)	1,134 (16.8)	55 (14.7)	
Heavy	448 (6.3)	424 (6.2)	24 (6.3)	
Skeletal muscle mass, median (IQR), kg	27.0 (20.2,32.8)	27.6 (20.6,33.0)	15.9 (14.3,23.3)	< 0.001
Skeletal muscle index, median (IQR), kg/m^2^	9.3 (7.6,10.5)	9.39 (7.7,10.6)	6.0 (5.7, 7.9)	< 0.001

IQR, interquartile range; CRP, C-reactive protein.

### 3.1 Cohort 1: daily alcohol consumption and sarcopenia

**Figure 2:**
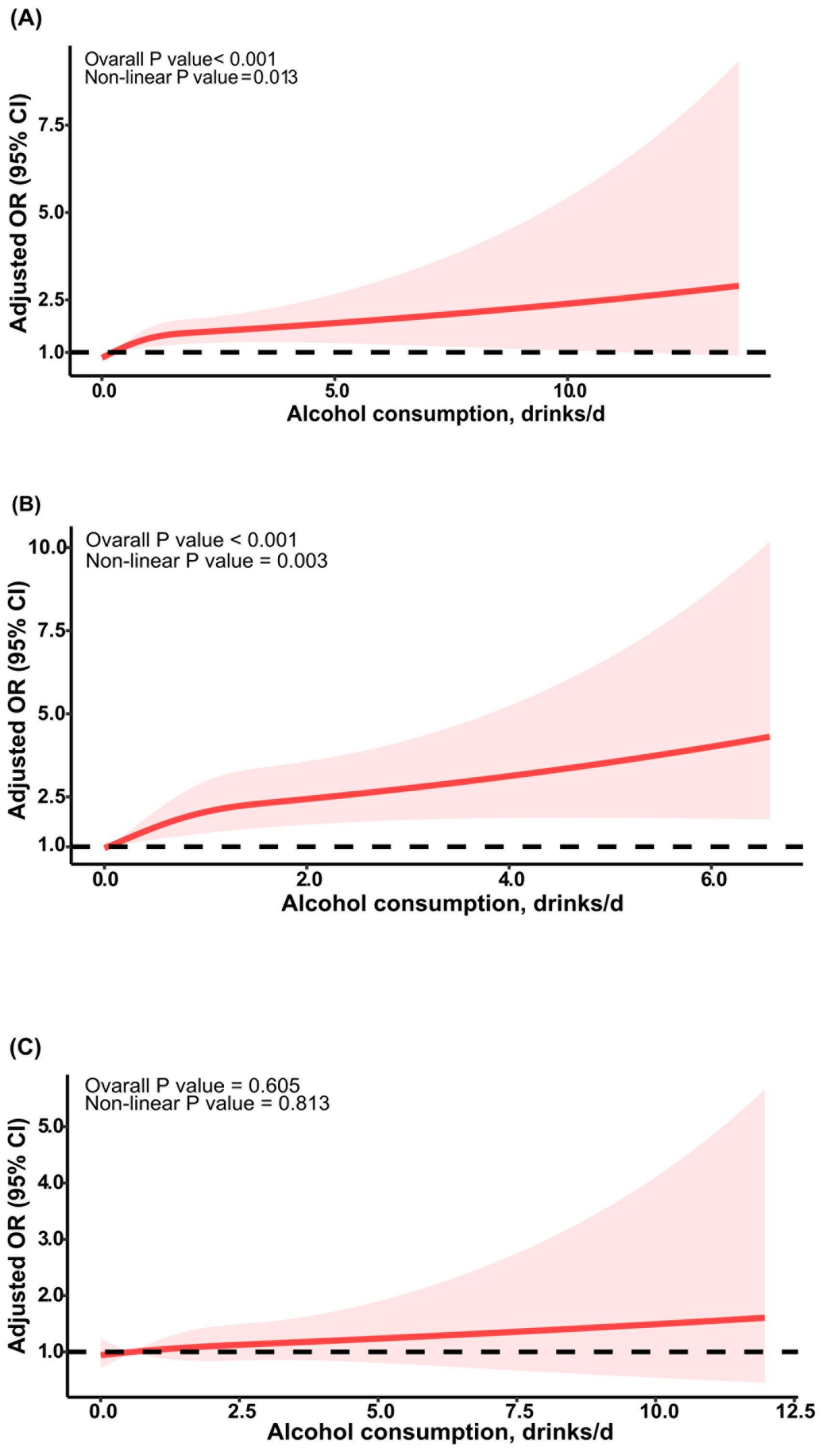
RCS curve of the association between alcohol consumption and sarcopenia. The association was adjusted for age, sex, ethnicity, education level, and marital status. **(A)** RCS curve for all participants; **(B)** RCS curve for female participants; **(C)** RCS curve for male participants. RCS, restricted cubic spline; OR, odds ratio.

Analysis of the continuous relationship between the number of drinks per day and the risk of developing sarcopenia was conducted using a restricted cubic spline regression model (Figure 2). We discovered a significant nonlinear dose–response relationship between the number of drinks per day and sarcopenia in all participants after adjusting for all confounding variables (*p*_overall_ < 0.001, *p*_non-linear_ = 0.01) (Figure 2A). A similar dose–response relationship was observed in females (*p*_overall_ < 0.001, *p*_non-linear_ < 0.01) (Figure 2B), but in males, the nonlinear relationship was not significant (*p*_overall_ = 0.60, *p*_non-linear_ = 0.81) (Figure 2C).

In all of the participants, drinking an additional drink of alcohol per day was associated with a 13% increase in the risk of developing sarcopenia in the age- and sex-adjusted model (OR: 1.13; 95% CI: 1.04–1.23) and the full model (OR: 1.13; 95% CI: 1.04–1.23) (Table 2). This association was accompanied by a higher risk of sarcopenia in females (full model: OR: 1.28; 95% CI: 1.10–1.48) but not in males (full model: OR: 1.05; 95% CI: 0.96–1.15) (Tables 3, 4). Additionally, the associations between the number of drinks per day and sarcopenia were consistent among other subgroups (Supplementary Table S4).

**Table 2:** Association between alcohol consumption and sarcopenia in all participants.

Group	No. of participants	No. of events	OR (95% CI)			*P* for trend
			Unadjusted	Age- and sex-adjusted model	Full model*	
No. of drinks, per day	7,592	446	1.01 (0.91,1.13)	1.13 (1.04,1.23)	1.13 (1.04,1.23)	
Alcohol drinking levels, per day						0.006
Nondrinkers	2,144	182	1	1	1	
Mild	3,811	185	0.57 (0.43,0.77)	1.51 (1.02,2.24)	1.65 (1.08,2.51)	
Moderate	1,189	55	0.58 (0.38,0.87)	1.87 (1.04,3.34)	2.04 (1.12,3.71)	
Heavy	448	24	0.67 (0.35,1.28)	2.30 (1.12,4.73)	2.42 (1.17,4.97)	
Drinking history						0.009
Nondrinkers	2,144	182	1	1	1	
Former drinker	4,285	331	0.94 (0.71,1.23)	1.18 (0.85,1.62)	1.21 (0.88,1.66)	
Current drinker	5,631	271	0.57 (0.43,0.77)	1.61 (1.07,2.42)	1.69 (1.12,2.56)	
No. of drinking days, per week	7,608	447	1.09 (1.02,1.17)	1.07 (1.00,1.14)	1.08 (1.01,1.15)	
Alcohol drinking frequency, per week						0.010
Nondrinkers	2,144	182	1	1	1	
1 day	2,842	106	0.42 (0.30,0.61)	1.35 (0.86,2.12)	1.46 (0.89,2.39)	
2 days	1,016	44	0.67 (0.43,1.05)	2.17 (1.31,3.61)	2.36 (1.40,3.99)	
>2 days	1,606	115	0.80 (0.56,1.14)	1.66 (1.01,2.74)	1.84 (1.10,3.07)	
No. of binge drinking days, per month	7,469	443	0.97 (0.92,1.01)	1.01 (0.99,1.04)	1.01 (0.99,1.04)	
Binge drinking frequency, per month						0.012
Nondrinkers	2,144	182	1	1	1	
≤ 1 day	3,866	218	0.64 (0.48,0.86)	1.55 (1.03,2.34)	1.68 (1.09,2.60)	
> 1 day	1,639	43	0.37 (0.21,0.64)	2.02 (1.06,3.86)	2.10 (1.10,4.01)	

*Weighted logistic regression adjusted for age, sex, ethnicity, education level, and marital status, with these variables determined by the directed acyclic graphs (DAGs). OR, odds ratio; CI, Confidence Interval.

**Table 3:** Association between alcohol consumption and sarcopenia in females.

Group	No. of participants	No. of events	OR (95% CI)			P for trend
			Unadjusted	Age-adjusted model	Full model*	
No. of drinks, per day	3,603	254	1.17 (1.00,1.37)	1.27 (1.10,1.48)	1.28 (1.10,1.48)	
Alcohol drinking levels, per day						<0.001
Nondrinkers	1,643	141	1	1	1	
Mild	1,640	90	0.68 (0.49,0.94)	1.86 (1.18,2.92)	2.11 (1.30,3.42)	
Moderate	224	18	1.23 (0.70,2.17)	2.97 (1.41,6.28)	3.42 (1.61,7.27)	
Heavy	96	5	0.81 (0.29,2.28)	2.39 (0.66,8.60)	2.55 (0.70,9.28)	
Drinking history						0.001
Nondrinkers	1,643	141	1	1	1	
Former drinker	2,477	175	0.94 (0.69,1.26)	1.21 (0.83,1.76)	1.24 (0.85,1.81)	
Current drinker	2002	115	0.75 (0.54,1.03)	2.07 (1.31,3.28)	2.17 (1.36,3.44)	
No. of drinking days, per week	3,607	254	1.19 (1.09,1.31)	1.13 (1.03,1.23)	1.14 (1.04,1.24)	
Alcohol drinking frequency, per week						0.002
Nondrinkers	1,643	141	1	1	1	
1 day	1,218	43	0.48 (0.29,0.79)	1.65 (0.90,3.02)	1.85 (0.99,3.47)	
2 days	348	24	0.99 (0.53,1.82)	2.85 (1.26,6.44)	3.25 (1.38,7.67)	
>2 days	398	46	1.35 (0.86,2.13)	2.11 (1.18,3.79)	2.44 (1.35,4.41)	
No. of binge drinking days, per month	3,617	255	0.98 (0.87,1.10)	1.02 (0.95,1.10)	1.02 (0.95,1.09)	
Binge drinking frequency, per month						0.003
Nondrinkers	1,463	141	1	1	1	
≤ 1 day	1,677	107	0.79 (0.57,1.08)	1.99 (1.27,3.12)	2.25 (1.39,3.63)	
> 1 day	297	7	0.49 (0.17,1.41)	2.37 (0.67,8.39)	2.69 (0.75,9.65)	

*Weighted logistic regression adjusted for age, ethnicity, education level, and marital status, with these variables determined by the directed acyclic graphs (DAGs). OR, odds ratio; CI, Confidence Interval.

**Table 4:** Association between alcohol consumption and sarcopenia in males.

Group	No. of participants	No. of events	OR (95% CI)			P for trend
			Unadjusted	Age-adjusted model	Full model*	
No. of drinks, per day	3,989	192	1.05 (0.95,1.15)	1.06 (0.97,1.17)	1.05 (0.96,1.15)	
Alcohol drinking levels, per day						0.542
Nondrinkers	501	41	1	1	1	
Mild	2,171	95	0.64 (0.36,1.15)	0.88 (0.46,1.66)	0.96 (0.50,1.85)	
Moderate	965	37	0.56 (0.28,1.11)	0.86 (0.39,1.88)	0.89 (0.40,1.96)	
Heavy	352	19	0.96 (0.39,2.39)	1.41 (0.55,3.63)	1.45 (0.56,3.79)	
Drinking history						0.969
Nondrinkers	501	41	1	1	1	
Former drinker	1808	156	1.20 (0.72,1.99)	0.97 (0.58,1.62)	1.01 (0.61,1.70)	
Current drinker	3,629	156	0.64 (0.36,1.13)	0.96 (0.51,1.80)	1.02 (0.53,1.94)	
No. of drinking days, per week	4,001	193	1.08 (1.00,1.17)	1.06 (1.04,1.08)	1.01 (0.94,1.08)	
Alcohol drinking frequency, per week						0.686
Nondrinkers	501	41	1	1	1	
1 day	1,624	63	0.52 (0.28,0.96)	0.82 (0.41,1.65)	0.88 (0.43,1.80)	
2 days	668	20	0.66 (0.31,1.37)	1.13 (0.49,2.62)	1.23 (0.52,2.93)	
>2 days	1,208	69	0.83 (0.43,1.59)	0.94 (0.47,1.91)	1.01 (0.50,2.04)	
No. of binge drinking days, per month	4,032	188	0.99 (0.96,1.03)	1.01 (0.98,1.04)	1.01 (0.98,1.03)	
Binge drinking frequency, per month						0.664
Nondrinkers	501	41	1	1	1	
≤ 1 day	2,189	111	0.69 (0.39,1.21)	0.83 (0.44,1.57)	0.90 (0.46,1.73)	
> 1 day	1,342	36	0.52 (0.27,1.00)	1.09 (0.52,2.31)	1.10 (0.52,2.34)	

*Weighted logistic regression adjusted for age, ethnicity, education level, and marital status, with these variables determined by the directed acyclic graphs (DAGs). OR, odds ratio; CI, Confidence Interval.

When classified by daily drinking level, the risk of sarcopenia was significantly associated with mild (OR: 1.65; 95% CI: 1.08–2.51), moderate (OR: 2.04; 95% CI: 1.12–3.71), and heavy drinker (OR: 2.42; 95% CI: 1.17–4.97) compared to nondrinkers, after adjusting for all variables. Moreover, these associations exhibited a linear trend (*p*_trend_ = 0.006) (Table 2). For females, similar associations were observed in mild (OR: 2.11; 95% CI: 1.30–3.42) and moderate drinkers (OR: 3.42; 95% CI: 1.61–7.27), but in heavy drinkers, it did not reach statistical significance (OR: 2.55; 95% CI: 0.70–9.28) (Table 3). For males, these associations and linear trends were not statistically significant (Table 4).

The sensitivity analysis, defining sarcopenia by low SMI and low gait speed, revealed that the association between quantitative daily alcohol consumption and sarcopenia did not achieve statistical significance in the overall participants, males, or females. The association between daily drinking level and sarcopenia in all participants was consistent with the main analysis, but it did not reach statistical significance in heavy drinkers (Supplementary Table S5). These associations were similar to the main analysis in males and females (Supplementary Tables S6, S7). In another sensitivity analysis adjusting for additional variables, the results were consistent with the main analysis (Supplementary Table S8).

### 3.2 Cohort 2: drinking history and sarcopenia

In both the age- and sex-adjusted model and the full model, the risk of developing sarcopenia was significantly higher in current drinkers (OR: 1.61; 95% CI: 1.07–2.42; 1.69; 95% CI: 1.12–2.56) compared to nondrinkers, whereas no significant difference was observed for former drinkers (OR: 1.18; 95% CI: 0.85–1.62; 1.21; 95% CI: 0.88–1.66) (Table 2). Similar results were found in female participants, while in males, these associations were not statistically significant (Tables 3, 4).

The sensitivity analysis based on the different definitions of sarcopenia showed that both former (full model: OR: 1.71; 95% CI: 1.16–2.53) and current drinkers (full model: OR: 2.01; 95% CI: 1.29–3.15) had a significantly increased risk of sarcopenia compared to nondrinkers in all participants (Supplementary Table S5). These associations in females were similar to all participants (Supplementary Table S6). In male participants, only former drinkers had an increased risk of sarcopenia (Supplementary Table S7). The linear trend test was significant in all and female participants, regardless of which definition was adopted (Supplementary Tables S5, S6). The results from another sensitivity analysis, which adjusted for additional variables, were consistent with the main analysis (Supplementary Table S8).

### 3.3 Cohort 3: drinking days per week and sarcopenia

When considered a quantitative variable, each additional day of alcohol consumption per week was associated with an approximately 8% increased risk of sarcopenia, regardless of whether it was in the unadjusted model (OR: 1.09; 95% CI: 1.02–1.17), age- and sex-adjusted model (OR: 1.07; 95% CI: 1.00–1.14), or full model (OR: 1.08; 95% CI: 1.01–1.15) (Table 2). The increased risk of sarcopenia was also observed in females with each additional day of alcohol consumption, but not in males after adjusting for the full variables (Tables 3, 4).

When the number of drinking days per week was categorized, compared to nondrinkers, the risk of developing sarcopenia did not significantly increase for those who drank 1 day a week (full model: OR: 1.46; 95% CI: 0.89–2.39), whereas the risk was significantly increased for drinkers who consumed alcohol 2 days (full model: OR: 2.36; 95% CI: 1.40–3.99) or more (full model: OR: 1.84; 95% CI: 1.10–3.07) per week in all participants (Table 2). Similar results were found in females but not males (Tables 3, 4).

In the sensitivity analysis using the alternative definition of sarcopenia, consistent results were observed regarding the association between each additional day of alcohol consumption and the risk of sarcopenia; the risk of sarcopenia was significantly higher among all participants who consumed alcohol 1, 2, or > 2 days per week compared to nondrinkers (Supplementary Table S5). In female participants, the association between the risk of sarcopenia and drinking alcohol 2 days per week did not reach statistical significance (Supplementary Table S6). In male participants, the frequency of weekly alcohol consumption was not significantly associated with the risk of sarcopenia (Supplementary Table S7). In another sensitivity analysis adjusting for additional variables, the results were consistent with the main analysis (Supplementary Table S8).

### 3.4 Cohort 4: binge drinking days per month and sarcopenia

The risk of developing sarcopenia was not significantly associated with each additional day of binge drinking per month in all participants, females, or males. In the full model, compared to nondrinkers, participants who engaged in binge drinking for ≤1 day per month (OR: 1.68; 95% CI: 1.09–2.60) or > 1 day per month (OR: 2.10; 95% CI: 1.10–4.01) had a significantly higher risk of developing sarcopenia (Table 2). For females, the risk of sarcopenia was also increased in participants with binge drinking frequency of ≤1 day per month (full model: OR: 2.25; 95% CI: 1.39–3.63), but lost statistical significance in those with a frequency of >1 day (full model: OR: 2.69; 95% CI: 0.75–9.65) (Table 3). For males, there was no significant association between binge drinking frequency per month and the occurrence of sarcopenia (Table 4).

The two sensitivity analyses yielded consistent results regarding the association between the frequency of binge drinking per month and the risk of sarcopenia, both in quantitative analysis and stratified analyses, among all participants as well as male or female participants (Supplementary Tables S5–S8).

## 4 Discussion

In this study, we found that one additional drink per day was independently associated with the occurrence of sarcopenia. Hierarchically, mild, moderate, and heavy daily drinking were associated with an increased risk of sarcopenia compared to nondrinkers. In terms of alcohol history, current drinkers were associated with a higher risk of sarcopenia. Former drinkers did not appear to be associated with the occurrence of sarcopenia; however, this finding was not supported by sensitivity analysis using alternative definitions of sarcopenia. The number of drinking days per week was associated with the risk of sarcopenia, with the risk being higher among participants who consumed alcohol on 1, 2, or more days per week compared to nondrinkers. Participants who engaged in binge drinking for ≤1 day per month or > 1 day per month had a significantly higher risk of developing sarcopenia compared to nondrinkers. Additionally, these associations exhibited sex differences, with female participants showing results largely consistent with the overall population, whereas male participants showed no significant differences. Sensitivity analyses confirmed the association between alcohol consumption and sarcopenia, as well as the sex differences in this association.

This study found that the overall prevalence of sarcopenia was approximately 6.0%, with a higher prevalence in females compared to males (8.0% vs. 4.3%). Studies on sarcopenia involving subjects under 60 years of age are rare. Our study reported a lower prevalence of sarcopenia compared to other studies on the general elderly population, which is likely due to the inclusion of relatively younger participants, considering the age-dependent nature of sarcopenia (5, 6). A projection model based on data from the populations of 28 European Union countries suggested that the number of sarcopenic patients would increase dramatically by 63.8% over the next 30 years, making sarcopenia a continuing major public health issue (56). The potential association between chronic excessive alcohol consumption and risk of sarcopenia is multifactorial. Evidence from cellular and animal studies suggests that excessive alcohol intake exacerbates the risk of sarcopenia through direct and indirect mechanisms related to impaired skeletal muscle protein metabolism, (16). Chronic excessive alcohol intake may lead to gut microbiota dysbiosis, autophagy-induced hyperammonemia, increased intestinal permeability, and elevated circulating endotoxins (16). The latter two may progressively contribute to subsequent systemic inflammation and insulin resistance. These changes lead to the activation of myostatin, AMPK and REDD1, along with the deactivation of insulin-like growth factor-1 (IGF-1), which in turn trigger the upregulation of muscle protein breakdown and the downregulation of muscle protein synthesis (16). Interestingly, previous studies have reported the beneficial effects of alcohol withdrawal on improving gut microbiota dysbiosis, gut permeability, and microbial translocation (57, 58). This suggests that alcohol abstinence may be beneficial in mitigating sarcopenia. In our main analysis, former drinkers appeared to be unrelated to the occurrence of sarcopenia; however, this finding was not supported by the sensitivity analysis using alternative definitions of sarcopenia. The value of alcohol abstinence in sarcopenia warrants further investigation in future studies.

There is currently significant controversy regarding the association between alcohol consumption and sarcopenia. A Filipino study involving 164 outpatient patients aged 40 and older found that alcoholic beverage drinking was significantly associated with developing sarcopenia compared to nondrinkers (18). A study involving 542 community-dwelling Singaporeans showed that alcoholism was significantly associated with sarcopenia (19). Another study with 2,176 US participants younger than 65 years found that alcohol drinking had a significant adverse effect on sarcopenia compared to the healthy behavior group (56). However, other studies have found no significant association between alcohol consumption and sarcopenia (20–22). In addition, two meta-analyses assessing the association between alcohol consumption and sarcopenia yielded different conclusions. One meta-analysis suggested that alcohol consumption may decrease the risk of sarcopenia (25), whereas the other more recent meta-analysis concluded that alcohol consumption is not associated with an increased risk of developing sarcopenia (26). Previous studies have generally included smaller sample sizes, and their assessments were primarily focused on older populations or in Asian regions. More importantly, none of these studies had the primary objective of investigating the association between alcohol intake and sarcopenia, which resulted in a lack of objectivity and comprehensiveness in defining alcohol exposure. Our study included a larger sample size and comprehensively assessed the association of drinking behavior, including drinking level, drinking history, weekly frequency of drinking, and monthly frequency of binge drinking, with sarcopenia in a nationally representative US cohort involving a multiracial and multiage population.

Our study found that alcohol consumption significantly increased the risk of sarcopenia, and further stratified analysis suggested that this association was primarily seen in female participants. These results were consistent with the findings of Han et al. (59), who found that daily alcohol consumption significantly increased the risk of sarcopenia in females but not males. Two other studies also showed a significant association between alcohol intake and sarcopenia in the female population (60, 61). Additionally, the overall findings of this investigation align with the existing literature, which suggests that women are more susceptible to adverse health effects related to alcohol consumption than men. Women drinkers are more prone to developing alcoholic liver disease, liver cirrhosis, and alcoholic hepatitis even when consuming less alcohol and for shorter periods of alcohol use compared to men (29). Similarly, women are more susceptible to alcohol-related heart disease or cardiomyopathy and stroke than men at the same or lower drinking volumes (30). Women who engage in moderate drinking show increased risk of cancer, whereas men must drink more to show the same increased risk (31). For the same alcohol intake, women develop higher blood alcohol levels compared to men. This is attributed to their lower *χ*-alcohol dehydrogenase activity in the stomach, which reduces first-pass metabolism and increases ethanol bioavailability (28). Women have lower body water content per kilogram of body weight, resulting in a smaller volume of ethanol distribution. Additionally, women have a slower rate of gastric emptying of alcohol (28). These factors contribute to higher blood alcohol levels and greater vulnerability of women to the pharmacologic effects of ethanol on skeletal muscle protein metabolism. Growth hormone (GH)/IGF-1 promotes muscle protein synthesis primarily through the PI3K/Akt/mTOR pathway. Additionally, PI3K/Akt inhibits FoxOs, thereby suppressing the transcription of E3 ubiquitin ligases that regulate protein degradation through the ubiquitin-proteasome system (62). Previous studies have confirmed the inhibitory effects of ethanol on the GH/IGF-1 axis (63). A weaker alcohol metabolism in women leads to a more pronounced impact of alcohol-related toxicity on the GH/IGF-1 axis compared to men. This was supported by a study that found a more significant decrease in IGF-1 levels after alcohol consumption in elderly women (64). Chronic inflammation is one of the core mechanisms underlying the development of sarcopenia. An animal study showed that chronic-binge alcohol feeding resulted in higher circulating endotoxin levels in female mice compared to male mice (65). This may be another reason for the gender differences in the association between alcohol intake and sarcopenia.

The strength of this study lies in its large and diverse sample from Western population, encompassing individuals of various ages and ethnicities, as well as its multifaceted approach to defining alcohol exposure. This study has several limitations. First, the cross-sectional nature of the study prevented us from establishing a definitive causal relationship between alcohol and sarcopenia. Nonetheless, the probability of increased alcohol consumption due to sarcopenia is relatively low, which may mitigate the risk of reverse causation. Second, although the use of the DAG approach can assist in selecting rational covariates to minimize the magnitude of bias in the estimate, some potential factors (such as creatine and omega-3 fatty acids intake) could not be analyzed due to the unavailability of data, which may lead to the presence of potential residual confounding. Third, alcohol consumption was assessed using a questionnaire at a single point in time, which could introduce potential recall bias. However, we observed consistent results across multiple definitions of alcohol exposure, which may mitigate this bias. Fourth, due to the simultaneous stratification of participants by sex and drinking habits, the power may have been insufficient to accurately estimate the risk of drinking. Fifth, sarcopenia was defined using BIA-estimated SMM. Several guidelines have proposed defining sarcopenia as the presence of both low muscle mass and low muscle function. Our sensitivity analysis using this definition yielded consistent results with the main analysis. However, SMM measurement based on computed tomography/magnetic resonance imaging is more objective and accurate, and our results need to be further validated by future studies. Finally, our study utilized the NHANES III data, and the characteristics of the past population may differ from those of the current population, which could limit the generalizability of our findings, highlighting the necessity of validating our results in new populations.

In conclusion, this large study of US adults showed that alcohol consumption was associated with an increased risk of sarcopenia across all individuals. Notably, this association revealed a significant sex-specific difference, with a significant risk increase observed in females but not in males. From a sarcopenia prevention perspective, these findings support the notion that alcohol abstinence may promote muscle health, particularly in females. Large-scale cohort studies with long-term follow-up are needed to corroborate our findings.

## Data Availability

Publicly available datasets were analyzed in this study. This data can be found here: https://www.cdc.gov/nchs/nhanes/index.htm.

## Footnotes

^1^ https://wwwn.cdc.gov/nchs/nhanes/nhanes3/default.aspx
