# Supplementary material for: Sex disparity in the association between alcohol consumption and sarcopenia: a population-based study
Source: Front Nutr. 2025 Feb 7;12:1536488. doi: 10.3389/fnut.2025.1536488 (PMC11842256; doi:10.3389/fnut.2025.1536488)
Supplement: Supplementary file 1 [file Table_1.DOCX]

**Online Supplemental Data**

**Content**

**Supplementary Table 1.** Baseline characteristics of the study population (cohort 2) according to sarcopenia status.

**Supplementary Table 2.** Baseline characteristics of the study population (cohort 3) according to sarcopenia status.

**Supplementary Table 3.** Baseline characteristics of the study population (cohort 4) according to sarcopenia status.

**Supplementary Table 4**. Subgroup analyses on the association between the number of drinks (each additional drink) per day and sarcopenia.

**Supplementary Table 5.** Association between alcohol consumption and sarcopenia (defined by low muscle mass and low gait speed) in all participants.

**Supplementary Table 6.** Association between alcohol consumption and sarcopenia (defined by low muscle mass and low gait speed) in females.

**Supplementary Table 7.** Association between alcohol consumption and sarcopenia (defined by low muscle mass and low gait speed) in males.

**Supplementary Table 8.** Sensitivity analysis assessing the association between alcohol consumption and sarcopenia by additional adjustments.

**Supplementary Figure 1.** Directed acyclic graph (DAG) for the association between alcohol consumption and sarcopenia.

**Supplementary Table 1.** Baseline characteristics of the study population (cohort 2) according to sarcopenia status.

| **Variable** | **Total (n=12060)** | **Nonsarcopeinia (n=11276)** | **Sarcopenia**  **(n=784)** | | **P value** |
| --- | --- | --- | --- | --- | --- |
| **Estimate U.S population (N)** | 133847934 | 127573109 | 6274823 | |  |
| **Age, median (IQR), y** | 40(30,56) | 39(29,54) | 68(55,77) | | < 0.001 |
| **Sex, %** |  |  |  | < 0.001 | |
| Female | 50.6(45.8,55.4) | 49.8(48.9,50.7) | 66.1(61.4,70.9) |  | |
| Male | 49.4(45.1,53.7) | 50.2(49.3,51.1) | 33.9(29.1,38.6) |  | |
| **Ethnicity, %** |  |  |  | 0.010 | |
| Mexican-American | 4.8(3.9, 5.7) | 5.0(4.0,5.9) | 2.1(1.4,2.9) |  | |
| Non-Hispanic black | 10.5(9.1,11.9) | 10.6(9.2,12.0) | 9.5(7.2,11.8) |  | |
| Non-Hispanic white | 77.4(68.8,86.1) | 77.1(74.6,79.7) | 83.8(80.0,87.6) |  | |
| Other | 7.2(5.9, 8.6) | 7.3(5.8,8.9) | 4.6(1.7,7.5) |  | |
| **Married, %** |  |  |  | < 0.001 | |
| No | 35.5(31.5,39.5) | 35.0(33.0,37.1) | 44.8(40.1,49.6) |  | |
| Yes | 64.5(58.7,70.3) | 65.0(62.9,67.0) | 55.2(50.4,59.9) |  | |
| **Education level, %** |  |  |  | < 0.001 | |
| High school or higher | 89.0(80.6,97.4) | 89.6(88.3,90.9) | 76.9(71.6,82.2) |  | |
| Less than high school | 11.0(9.6,12.4) | 10.4(9.2,11.7) | 23.1(17.8,28.4) |  | |
| **Smoking, %** |  |  |  | 0.090 | |
| No | 45.8(41.6,49.9) | 45.9(44.4,47.5) | 42.1(37.6,46.6) |  | |
| Yes | 54.2(48.9,59.6) | 54.1(52.5,55.6) | 57.9(53.4,62.4) |  | |
| **BMI, median (IQR), kg/m^2^** | 25.3(22.4,29.1) | 25.5(22.7,29.4) | 21.5(19.5,23.6) | < 0.001 | |
| **Poverty income ratio, median (IQR)** | 2.8(1.7,4.2) | 2.8(1.7,4.2) | 2.5(1.4,4.0) | 0.050 | |
| **Energy intake, median (IQR), kcal/d** | 1998(1477,2763) | 2027(1498,2790) | 1541(1158,2077) | < 0.001 | |
| **Protein intake, median (IQR), g/d** | 73.0(51.8,100.9) | 73.9(52.3,101.6) | 59.8(42.9, 81.2) | < 0.001 | |
| **Vitamin D levels, median (IQR), nmol/l** | 61.5(46.0,78.8) | 61.8(46.2,79.0) | 59.0(43.9,76.7) | 0.020 | |
| **Total polyunsaturated fatty acids intake (g/d)** | 14.8( 8.7,23.4) | 15.0(8.8,23.8) | 10.8(6.7,17.5) | < 0.001 | |
| **Zinc** **intake (mg/d)** | 10.0( 6.7,14.5) | 10.1(6.8,14.7) | 8.2(5.5,11.6) | < 0.001 | |
| **CRP, median (IQR), mg/dl** | 0.2(0.2,0.3) | 0.2(0.2,0.3) | 0.2(0.2,0.4) | 0.010 | |
| **Physical activity level, %** |  |  |  | < 0.001 | |
| Inactive | 14.2(12.5,15.9) | 13.6(12.1,15.2) | 26.9(21.9,31.9) |  | |
| Moderate | 44.4(40.0,48.7) | 44.7(42.5,46.9) | 37.0(32.0,42.0) |  | |
| Active | 41.4(36.2,46.6) | 41.7(38.8,44.6) | 36.1(30.9,41.2) |  | |
| **Number of comorbidities, %** |  |  |  | < 0.001 | |
| 0-1 | 47.8(43.0,52.6) | 49.1(47.3,51.0) | 20.9(16.8,25.0) |  | |
| ≥2 | 52.2(47.4,57.1) | 50.9(49.1,52.8) | 79.1(75.0,83.2) |  | |
| **Drinking history, %** |  |  |  | < 0.001 | |
| Nondrinkers | 12.9(11.2,14.6) | 12.7(11.3,14.1) | 17.2(13.1,21.2) |  | |
| Former | 31.8(28.9,34.7) | 31.4(29.2,33.6) | 39.7(34.9,44.5) |  | |
| Current | 55.3(48.6,62.0) | 55.9(52.7,59.0) | 43.2(37.2,49.1) |  | |
| **Skeletal muscle mass, median (IQR), kg** | 25.6(20.0,32.1) | 26.3(20.3,32.4) | 15.7(14.1,22.8) | < 0.001 | |
| **Skeletal muscle index, median (IQR), kg/m^2^** | 9.0(7.6,10.5) | 9.2(7.7,10.5) | 6.0(5.6, 7.8) | < 0.001 | |

BMI, body mass index; CRP, C-reactive protein; IQR, interquartile range.

**Supplementary Table 2.** Baseline characteristics of the study population (cohort 3) according to sarcopenia status.

| **Variable** | **Total (n=7608)** | **Nonsarcopeinia**  **(n=7161)** | **Sarcopenia(n=447)** | **P value** |
| --- | --- | --- | --- | --- |
| **Estimate U.S population (N)** | 89997373 | 86225665 | 3771708 |  |
| **Age, median (IQR), y** | 38(28,52) | 37(28,50) | 64(50,75) | < 0.001 |
| **Sex, %** |  |  |  | < 0.001 |
| Female | 46.5(41.0,51.9) | 45.7(44.2,47.1) | 64.9(58.8,70.9) |  |
| Male | 53.6(48.0,59.1) | 54.4(53.0,55.8) | 35.2(29.1,41.2) |  |
| **Ethnicity, %** |  |  |  | 0.070 |
| Mexican-American | 4.8(3.9, 5.7) | 4.9(3.9,5.9) | 2.2(1.3,3.1) |  |
| Non-Hispanic black | 9.9(8.7,11.2) | 10.0(8.5,11.4) | 9.0(6.611.5) |  |
| Non-Hispanic white | 77.3(67.1,87.5) | 77.1(74.3,79.8) | 83.3(78.6,87.9) |  |
| Others | 8.0(6.3, 9.6) | 8.1(6.3,9.9) | 5.5(1.8,9.3) |  |
| **Married, %** |  |  |  | 0.110 |
| No | 37.3(32.5,42.0) | 37.0(34.7,39.4) | 42.7(36.0,49.4) |  |
| Yes | 62.7(55.9,69.5) | 63.0(60.7,65.3) | 57.3(50.6,64.0) |  |
| **Education level, %** |  |  |  | < 0.001 |
| High school or higher | 90.6(80.3,100.8) | 91.1(89.7,92.4) | 79.4(73.3,85.5) |  |
| Less than high school | 9.4(8.2, 10.7) | 9.0(7.6,10.3) | 20.6(14.6,26.7) |  |
| **Smoking, %** |  |  |  | 0.140 |
| No | 47.2(42.0,52.4) | 47.3(45.6,49.1) | 43.3(37.8,48.8) |  |
| Yes | 52.8(46.9,58.8) | 52.7(50.9,54.4) | 56.7(51.2,62.2) |  |
| **BMI, median (IQR), kg/m^2^** | 25.0(22.2,28.5) | 25.1(22.4,28.6) | 21.5(19.2,23.8) | < 0.001 |
| **Poverty income ratio, median (IQR)** | 3.0(1.8,4.4) | 3.0(1.8,4.4) | 2.8(1.6,4.5) | 0.500 |
| **Energy intake, median (IQR), kcal/d** | 2088(1537,2859) | 2114(1559,2882) | 1591(1200,2116) | < 0.001 |
| **Protein intake, median (IQR), g/d** | 75.2(54.0,103.9) | 76.2(54.4,104.8) | 61.6(43.9, 82.0) | < 0.001 |
| **Vitamin D levels, median (IQR), nmol/l** | 62.8(46.8,80.3) | 62.8(47.0,80.5) | 59.2(42.4,77.4) | 0.020 |
| **Total polyunsaturated fatty acids intake (g/d)** | 15.3(9.2,24.4) | 15.4(9.4,24.7) | 10.7(6.8,17.5) | < 0.001 |
| **Zinc** **intake (mg/d)** | 10.5(7.0,14.9) | 10.6(7.0,15.1) | 8.4(5.7,11.6) | < 0.001 |
| **CRP, median (IQR), mg/dl** | 0.2(0.2,0.2) | 0.2(0.2,0.2) | 0.2(0.2,0.4) | 0.060 |
| **Physical activity level, %** |  |  |  | < 0.001 |
| Inactive | 12.1(10.4,13.8) | 11.6(9.9,13.3) | 23.5(17.5,29.5) |  |
| Moderate | 44.4(39.5,49.3) | 44.5(41.9,47.1) | 41.5(35.2,47.9) |  |
| Active | 43.5(37.0,50.0) | 43.9(40.6,47.2) | 34.9(28.7,41.2) |  |
| **Number of comorbidities, %** |  |  |  | < 0.001 |
| 0-1 | 51.9(45.9,58.0) | 53.1(51.1,55.1) | 24.9(19.0,30.7) |  |
| ≥2 | 48.1(42.8,53.4) | 46.9(44.9,49.0) | 75.1(69.3,81.0) |  |
| **No. of drinking days per week, median (IQR)** | 1.0(0.2,2.0) | 1.0(0.2,2.0) | 1.0(0.0,3.9) | 0.870 |
| **Alcohol drinking frequency per week, %** |  |  |  | < 0.001 |
| Nondrinkers | 19.2(16.7,21.8) | 18.8(16.4,21.2) | 28.5(21.7,35.4) |  |
| One day | 41.4(36.6,46.3) | 42.1(39.7,44.4) | 27.1(21.0,33.2) |  |
| 2 days | 15.1(12.4,17.8) | 15.1(13.4,16.7) | 15.2(10.2,20.3) |  |
| >2 days | 24.3(20.7,27.9) | 24.1(22.1,26.1) | 29.1(22.5,35.7) |  |
| **Skeletal muscle mass, median (IQR), kg** | 27.0(20.2,32.8) | 27.6(20.6,33.0) | 15.9(14.3,23.3) | < 0.001 |
| **Skeletal muscle index, median (IQR), kg/m^2^** | 9.3(7.6,10.5) | 9.4(7.7,10.6) | 6.0(5.7, 7.9) | < 0.001 |

BMI, body mass index; CRP, C-reactive protein; IQR, interquartile range.

**Supplementary Table 3.** Baseline characteristics of the study population (cohort 4) according to sarcopenia status.

| **Variable** | **Total (n=7649)** | **Nonsarcopeinia**  **(n=7206)** | **Sarcopenia(n=443)** | **P value** |
| --- | --- | --- | --- | --- |
| **Estimate U.S population (N)** | 90563909 | 86826333 | 3737576 |  |
| **Age, median (IQR), y** | 38 (28,52) | 37(28,50) | 64(50,75) | < 0.001 |
| **Sex, %** |  |  |  | < 0.001 |
| Female | 46.2(40.8,51.6) | 45.4(44.0,46.8) | 65.5(59.3,71.8) |  |
| Male | 53.8(48.1,59.4) | 54.6(53.2,56.0) | 34.5(28.2,40.8) |  |
| **Ethnicity, %** |  |  |  | 0.070 |
| Mexican-American | 4.8(3.9, 5.7) | 4.9(3.9,5.9) | 2.2(1.3,3.1) |  |
| Non-Hispanic black | 9.9(8.6,11.2) | 9.9(8.5,11.4) | 9.0(6.5,11.4) |  |
| Non-Hispanic white | 77.4(67.1,87.7) | 77.2(74.4,80.0) | 83.6(78.9,88.4) |  |
| Others | 7.9(6.3, 9.5) | 8.0(6.2,9.8) | 5.2(1.4,9.0) |  |
| **Married, %** |  |  |  | 0.130 |
| No | 37.4(32.6,42.2) | 37.2(34.9,39.6) | 42.5(35.8,49.2) |  |
| Yes | 62.6(55.8,69.4) | 62.8(60.6,65.1) | 57.5(50.8,64.2) |  |
| **Education level, %** |  |  |  | < 0.001 |
| High school or higher | 90.6(80.3,101.0) | 91.1(89.9,92.4) | 79.4(73.4,85.5) |  |
| Less than high school | 9.4(8.1, 10.6) | 8.9(7.6,10.2) | 20.6(14.6,26.6) |  |
| **Smoking, %** |  |  |  | 0.180 |
| No | 47.2(41.9,52.4) | 47.3(45.6,49.0) | 43.7(38.2,49.1) |  |
| Yes | 52.9(46.8,58.9) | 52.7(51.0,54.4) | 56.3(50.9,61.8) |  |
| **BMI, median (IQR), kg/m^2^** | 25.0(22.3,28.5) | 25.1(22.4,28.7) | 21.5(19.2,23.8) | < 0.001 |
| **Poverty income ratio, median (IQR)** | 3.0(1.8,4.4) | 3.0(1.8,4.4) | 2.9(1.6,4.5) | 0.600 |
| **Energy intake, median (IQR), kcal/d** | 2088(1539,2862) | 2114(1559,2889) | 1591(1200,2115) | < 0.001 |
| **Protein intake, median (IQR), g/d** | 75.1(54.0,103.9) | 76.2(54.4,104.9) | 61.6(43.9, 82.0) | < 0.001 |
| **Vitamin D levels, median (IQR), nmol/l** | 62.8(47.0,80.3) | 62.8(47.3,80.5) | 59.7(43.5,77.4) | 0.030 |
| **Total polyunsaturated fatty acids intake (g/d)** | 15.2(9.2,24.3) | 15.4(9.4,24.6) | 10.7(6.8,17.5) | < 0.001 |
| **Zinc** **intake (mg/d)** | 10.5(7.0,14.9) | 10.6(7.1,15.1) | 8.4(5.7,11.6) | < 0.001 |
| **CRP, median (IQR), mg/dl** | 0.2(0.2,0.2) | 0.2(0.2,0.2) | 0.2(0.2,0.4) | 0.040 |
| **Physical activity level, %** |  |  |  | < 0.001 |
| Inactive | 12.1(10.4,13.9) | 11.7(9.9,13.4) | 22.9(17.0,28.9) |  |
| Moderate | 44.4(39.4,49.4) | 44.5(42.0,47.0) | 41.9(35.4,48.4) |  |
| Active | 43.5(37.0,49.9) | 43.8(40.6,47.0) | 35.2(29.0,41.3) |  |
| **Number of comorbidities, %** |  |  |  | < 0.001 |
| 0-1 | 52.1(46.1,58.2) | 53.3(51.2,55.3) | 25.2(19.4,31.0) |  |
| ≥2 | 47.9(42.5,53.3) | 46.7(44.7,48.8) | 74.8(69.0,80.6) |  |
| **No. of binge drinking days per month, median (IQR)** | 0(0,1) | 0(0,1) | 0(0,0) | < 0.001 |
| **Binge drinking levels pre month, %** |  |  |  | < 0.001 |
| Nondrinkers | 19.1(16.6,21.6) | 18.7(16.2,21.1) | 28.8(21.9,35.7) |  |
| ≤1 day | 59.4(51.9,66.9) | 59.4(56.6,62.2) | 58.8(52.6,65.5) |  |
| >1 day | 21.6(18.2,24.9) | 22.0(19.9,24.0) | 12.4(7.2,17.6) |  |
| **Skeletal muscle mass, median (IQR), kg** | 27.1(20.3,32.8) | 27.7(20.6,33.0) | 15.9(14.2,23.2) | < 0.001 |
| **Skeletal muscle index, median (IQR), kg/m^2^** | 9.3(7.7,10.5) | 9.4(7.7,10.6) | 6.0(5.7, 7.9) | < 0.001 |

BMI, body mass index; CRP, C-reactive protein; IQR, interquartile range.

**Supplementary Table 4**. Subgroup analyses on the association between the number of drinks (each additional drink) per day and sarcopenia

| **Subgroup** | **No. of participants** | **No. of events** | **OR (95% CI)** | | ***P* for interaction** |
| --- | --- | --- | --- | --- | --- |
|  |  |  | **Unadjusted** | **Full model*** |  |
| **Age** | | | | | 0.553 |
| ≤40 | 4557 | 48 | 0.96(0.72,1.27) | 0.94(0.70, 1.26) |  |
| > 40 | 3035 | 398 | 1.03(0.93,1.14) | 1.17(1.06,1.28) |  |
| **Ethnicity** |  |  |  |  | 0.523 |
| Non-Hispanic white | 3202 | 265 | 0.98(0.84,1.14) | 1.12(0.99,1.26) |  |
| Non-Hispanic black | 2030 | 95 | 1.13(1.05,1.22) | 1.17(1.09,1.27) |  |
| Mexican-American | 2047 | 70 | 0.83(0.55,1.24) | 1.09(0.86,1.39) |  |
| Others | 313 | 16 | 0.97(0.70,1.35) | 1.02(0.67,1.54) |  |
| **Education level** |  |  |  |  | 0.154 |
| High school or higher | 6036 | 288 | 1.05(0.95,1.17) | 1.15(1.05,1.27) |  |
| Less than high school | 1556 | 158 | 0.88(0.68,1.14) | 1.04(0.87,1.25) |  |
| **Married** |  |  |  |  | 0.871 |
| No | 3112 | 224 | 0.96(0.82,1.11) | 1.10(0.98,1.22) |  |
| Yes | 4480 | 222 | 1.06(0.91,1.23) | 1.14(1.01,1.30) |  |
| **Smoking** |  |  |  |  | 0.347 |
| No | 3912 | 216 | 0.69(0.47,1.02) | 1.05(0.86,1.27) |  |
| Yes | 3680 | 230 | 1.05(0.94,1.16) | 1.12(1.02,1.22) |  |
| **Physical activity level** |  |  |  |  | 0.151 |
| Inactive | 1392 | 141 | 1.09(0.95,1.25) | 1.23(1.06,1.43) |  |
| Moderate | 3196 | 163 | 0.89(0.72,1.11) | 1.01(0.89,1.14) |  |
| Active | 3004 | 142 | 1.03(0.92,1.14) | 1.11(1.01,1.23) |  |
| **Number of comorbidities** |  |  |  |  | 0.880 |
| 0-1 | 3693 | 97 | 0.99(0.78,1.24) | 1.11(0.96,1.28) |  |
| ≥2 | 3899 | 349 | 1.01(0.91,1.12) | 1.13(1.02,1.25) |  |
| **Poverty income ratio (PIR)** |  |  |  |  | 0.460 |
| PIR < 1.3 | 2139 | 126 | 1.01(0.90,1.14) | 1.12(1.05,1.20) |  |
| PIR≥1.3 | 4764 | 268 | 1.00(0.84,1.18) | 1.10(0.97,1.26) |  |
| **BMI** |  |  |  |  | 0.673 |
| BMI < 25 | 3324 | 349 | 1.00(0.90,1.12) | 1.10(1.02,1.20) |  |
| BMI≥25 | 4268 | 97 | 1.04(0.73,1.48) | 1.19(0.87,1.63) |  |
| **Energy intake meets requirements** |  |  |  |  | 0.885 |
| Yes | 3135 | 169 | 1.04(0.91,1.19) | 1.10(0.98,1.23) |  |
| No | 4255 | 265 | 0.97(0.85,1.12) | 1.15(1.02,1.29) |  |
| **Protein intake meets requirements** |  |  |  |  | 0.859 |
| Yes | 3702 | 211 | 1.01(0.85,1.20) | 1.16(1.03,1.31) |  |
| No | 3688 | 223 | 1.02(0.88,1.18) | 1.11(0.97,1.26) |  |
| **Vitamin D deficiency** |  |  |  |  | 0.424 |
| Yes | 3124 | 178 | 1.08(0.97,1.19) | 1.14(1.01,1.28) |  |
| No | 4134 | 239 | 0.98(0.81,1.18) | 1.15(1.02,1.30) |  |
| **CRP level** |  |  |  |  | 0.171 |
| CRP > 0.3 | 1979 | 136 | 1.09(0.95,1.24) | 1.20(1.03,1.38) |  |
| CRP≤0.3 | 5217 | 266 | 0.95(0.83,1.09) | 1.12(1.03,1.22) |  |

*Weighted logistic regression adjusted for age, sex, ethnicity, education level, and marital status, with these variables determined by the directed acyclic graphs (DAGs). Sex, ethnicity, education level, and marital status were not adjusted within their own subgroups. OR, odds ratio; CI, confidence interval; BMI, body mass index; CRP, C-reactive protein.

**Supplementary Table 5.** Association between alcohol consumption and sarcopenia (defined by low muscle mass and low gait speed) in all participants

| **Group** | **No. of participants** | **No. of events** | **OR (95% CI)** | | | **P for trend** |
| --- | --- | --- | --- | --- | --- | --- |
|  |  |  | **Unadjusted** | **Age- and sex-adjusted model** | **Full model*** |  |
| **No. of drinks, per day** | 1759 | 282 | 0.92(0.77,1.10) | 1.06(0.92,1.24) | 1.07(0.94,1.23) |  |
| **Alcohol drinking levels, per day** | | | | | | 0.004 |
| Nondrinkers | 757 | 135 | 1 | 1 | 1 |  |
| Mild | 727 | 112 | 1.11(0.75,1.65) | 2.04(1.28,3.26) | 2.47(1.52,4.02) |  |
| Moderate | 200 | 28 | 1.11(0.61,2.02) | 2.20(1.09,4.43) | 2.76(1.42,5.37) |  |
| Heavy | 75 | 7 | 0.59(0.16,2.17) | 1.94(0.54,6.91) | 2.32(0.67,7.98) |  |
| **Drinking history** | | | | | | 0.003 |
| Nondrinkers | 757 | 135 | 1 | 1 | 1 |  |
| Former drinker | 1593 | 253 | 1.24(0.87,1.77) | 1.64(1.09,2.45) | 1.71(1.16,2.53) |  |
| Current drinker | 1039 | 153 | 1.05(0.71,1.55) | 1.83(1.16,2.87) | 2.01(1.29,3.15) |  |
| **No. of drinking days, per week** | 1763 | 283 | 1.00(0.93,1.08) | 1.06(0.98,1.15) | 1.09(1.00,1.18) |  |
| **Alcohol drinking frequency, per week** | | | | | | 0.007 |
| Nondrinkers | 757 | 135 | 1 | 1 | 1 |  |
| One day | 409 | 59 | 1.07(0.61,1.87) | 2.13(1.21,3.75) | 2.48(1.41,4.36) |  |
| 2 days | 149 | 21 | 1.21(0.70,2.09) | 2.22(1.09,4.48) | 2.64(1.25,5.60) |  |
| >2 days | 448 | 68 | 1.01(0.62,1.64) | 1.97(1.10,3.51) | 2.47(1.40,4.36) |  |
| **No. of Binge drinking days, per month** | 1772 | 284 | 0.95(0.90,1.00) | 0.98(0.94,1.03) | 0.98(0.94,1.02) |  |
| **Binge drinking frequency, per month** | | | | | | <0.001 |
| Nondrinkers | 757 | 135 | 1 | 1 | 1 |  |
| ≤ 1 day | 850 | 134 | 1.11(0.77,1.62) | 2.04(1.29,3.22) | 2.50(1.56,4.00) |  |
| > 1 day | 165 | 15 | 0.66(0.28,1.58) | 2.15(0.87,5.32) | 2.37(1.01,5.54) |  |

*Weighted logistic regression adjusted for age, sex, ethnicity, education level, and marital status, with these variables determined by the directed acyclic graphs (DAGs). OR, odds ratio; CI, confidence interval.

**Supplementary Table 6.** Association between alcohol consumption and sarcopenia (defined by low muscle mass and low gait speed) in females.

| **Group** | **No. of participants** | **No. of events** | **OR (95% CI)** | | | **P for trend** |
| --- | --- | --- | --- | --- | --- | --- |
|  |  |  | **Unadjusted** | **Age-adjusted model** | **Full model*** |  |
| **No. of drinks, per day** | 904 | 175 | 1.15(0.86,1.56) | 1.29(0.93,1.78) | 1.35(0.97,1.88) |  |
| **Alcohol drinking levels, per day** | | | | | | <0.001 |
| Nondrinkers | 598 | 107 | 1 | 1 | 1 |  |
| Mild | 256 | 56 | 1.56(0.91,2.67) | 2.13(1.20, 3.77) | 2.67(1.45, 4.91) |  |
| Moderate | 41 | 10 | 1.98(0.92,4.27) | 2.91(1.29, 6.58) | 3.90(1.68, 9.05) |  |
| Heavy | 9 | 2 | 1.11(0.16,7.44) | 2.09(0.30,14.34) | 2.61(0.38,17.84) |  |
| **Drinking history** | | | | | | 0.001 |
| Nondrinkers | 598 | 107 | 1 | 1 | 1 |  |
| Former drinker | 784 | 132 | 1.34(0.86,2.10) | 1.57(0.97,2.53) | 1.62(1.00,2.63) |  |
| Current drinker | 315 | 69 | 1.59(1.00,2.54) | 2.19(1.32,3.61) | 2.38(1.43,3.96) |  |
| **No. of drinking days, per week** | 905 | 175 | 1.07(0.98,1.17) | 1.10(1.00,1.22) | 1.13(1.02,1.26) |  |
| **Alcohol drinking frequency, per week** | | | | | | 0.004 |
| Nondrinkers | 598 | 107 | 1 | 1 | 1 |  |
| One day | 135 | 27 | 1.61(0.77,3.38) | 2.41(1.15,5.06) | 2.87(1.36,6.02) |  |
| 2 days | 52 | 11 | 1.42(0.60,3.38) | 1.86(0.67,5.13) | 2.30(0.77,6.86) |  |
| >2 days | 120 | 30 | 1.69(0.99,2.88) | 2.29(1.25,4.17) | 3.01(1.57,5.78) |  |
| **No. of binge drinking days, per month** | 910 | 176 | 0.91(0.81,1.03) | 0.94(0.85,1.03) | 0.94(0.85,1.04) |  |
| **Binge drinking frequency, per month** | | | | | | 0.001 |
| Nondrinkers | 598 | 107 | 1 | 1 | 1 |  |
| ≤ 1 day | 291 | 66 | 1.64(1.01,2.66) | 2.26(1.33,3.86) | 2.84(1.61,5.03) |  |
| > 1 day | 21 | 3 | 0.91(0.22,3.71) | 1.52(0.34,6.79) | 1.98(0.41,9.64) |  |

*Weighted logistic regression adjusted for age, ethnicity, education level, and marital status, with these variables determined by the directed acyclic graphs (DAGs). OR, odds ratio; CI, confidence interval.

**Supplementary Table 7.** Association between alcohol consumption and sarcopenia (defined by low muscle mass and low gait speed) in males.

| **Group** | **No. of participants** | **No. of events** | **OR (95% CI)** | | | **P for trend** |
| --- | --- | --- | --- | --- | --- | --- |
|  |  |  | **Unadjusted** | **Age-adjusted model** | **Full model*** |  |
| **No. of drinks, per day** | 855 | 107 | 0.94(0.75,1.18) | 0.99(0.79,1.23) | 0.98(0.79, 1.20) |  |
| **Alcohol drinking levels, per day** | | | | | | 0.832 |
| Nondrinkers | 159 | 28 | 1 | 1 | 1 |  |
| Mild | 471 | 56 | 1.18(0.70,1.98) | 1.43(0.80,2.56) | 1.70(0.80, 3.65) |  |
| Moderate | 159 | 18 | 1.01(0.39,2.60) | 1.14(0.45,2.89) | 1.24(0.52, 2.95) |  |
| Heavy | 66 | 5 | 0.82(0.18,3.75) | 1.33(0.29,6.15) | 1.47(0.30, 7.13) |  |
| **Drinking history** | | | | | | 0.943 |
| Nondrinkers | 159 | 28 | 1 | 1 | 1 |  |
| Former drinker | 809 | 121 | 1.56(0.92,2.66) | 1.67(0.98,2.83) | 1.75(1.03,2.96) |  |
| Current drinker | 724 | 84 | 1.09(0.66,1.82) | 1.30(0.79,2.15) | 1.42(0.82,2.49) |  |
| **No. of drinking days, per week** | 858 | 108 | 0.98(0.89,1.09) | 0.99(0.89,1.09) | 1.00(0.91, 1.11) |  |
| **Alcohol drinking frequency, per week** | | | | | | 0.601 |
| Nondrinkers | 159 | 28 | 1 | 1 | 1 |  |
| One day | 274 | 32 | 1.08(0.58,2.01) | 1.34(0.69,2.59) | 1.52(0.68, 3.38) |  |
| 2 days | 97 | 10 | 1.58(0.61,4.13) | 2.18(0.78,6.14) | 2.53(0.97, 6.58) |  |
| >2 days | 328 | 38 | 1.01(0.50,2.01) | 1.21(0.62,2.35) | 1.40(0.68, 2.92) |  |
| **No. of binge drinking days, per month** | 862 | 108 | 0.98(0.93,1.03) | 0.99(0.95,1.04) | 0.99(0.94, 1.04) |  |
| **Binge drinking frequency, per month** | | | | | | 0.364 |
| Nondrinkers | 159 | 28 | 1 | 1 | 1 |  |
| ≤ 1 day | 559 | 68 | 1.09(0.67,1.77) | 1.28(0.75,2.17) | 1.55(0.77, 3.12) |  |
| > 1 day | 144 | 12 | 1.05(0.36,3.08) | 1.72(0.61,4.80) | 1.66(0.54, 5.12) |  |

*Weighted logistic regression adjusted for age, ethnicity, education level, and marital status, with these variables determined by the directed acyclic graphs (DAGs). OR, odds ratio; CI, confidence interval.

**Supplementary Table 8.** Sensitivity analysis assessing the association between alcohol consumption and sarcopenia with additional adjustments.

| **Group** | **Adjusted OR (95% CI)^*^** | | | |  |
| --- | --- | --- | --- | --- | --- |
|  | **All participants** | **Females** | **Males** | |  |
| **No. of drinks, per day** | 1.15(1.06,1.25) | 1.30(1.11,1.53) | 1.04(0.95,1.14) | |  |
| **Alcohol drinking levels, per day** | | | | |  |
| Nondrinkers | 1 | 1 | 1 | |  |
| Mild | 1.96(1.27,3.03) | 2.26(1.33, 3.85) | 1.39(0.77,2.51) | |  |
| Moderate | 2.42(1.37,4.28) | 2.93(1.34, 6.40) | 1.55(0.77,3.13) | |  |
| Heavy | 2.95(1.27,6.85) | 2.21(0.42,11.58) | 2.49(0.94,6.63) | |  |
| *P* for trend | 0.002 | 0.005 | 0.094 | |  |
| **Drinking history** | | | |  | |
| Nondrinkers | 1 | 1 | 1 | |  |
| Former drinker | 1.30(0.93,1.82) | 1.24(0.81,1.89) | 1.47(0.85,2.54) | |  |
| Current drinker | 1.91(1.25,2.91) | 2.24(1.39,3.60) | 1.49(0.83,2.68) | |  |
| *P* for trend | 0.002 | 0.001 | 0.442 | |  |
| **No. of drinking days, per week** | 1.10(1.02,1.18) | 1.12(1.02,1.24) | 1.01(0.94,1.09) | |  |
| **Alcohol drinking frequency, per week** | | | | |  |
| Nondrinkers | 1 | 1 | 1 | |  |
| One day | 1.69(0.99,2.89) | 2.00(1.01,3.98) | 1.21(0.63,2.33) | |  |
| 2 days | 2.94(1.76,4.91) | 3.49(1.49,8.18) | 1.95(0.92,4.12) | |  |
| >2 days | 2.25(1.29,3.91) | 2.37(1.21,4.63) | 1.70(0.85,3.39) | |  |
| *P* for trend | 0.004 | 0.005 | 0.123 | |  |
| **No. of binge drinking days, per month** | 1.02(0.99,1.05) | 1.03(0.96,1.11) | 1.00(0.97,1.03) | |  |
| **Binge drinking frequency, per month** | | | | |  |
| Nondrinkers | 1 | 1 | 1 | |  |
| ≤ 1 day | 1.94(1.24,3.03) | 2.29(1.35, 3.88) | 0.86(0.46,1.63) | |  |
| > 1 day | 2.88(1.44,5.74) | 2.94(0.84,10.29) | 1.06(0.50,2.25) | |  |
| *P* for trend | 0.001 | 0.003 | 0.729 | |  |

*Weighted logistic regression adjusted for age, sex, ethnicity, education level, marital status, daily protein intake, physical activity level, daily polyunsaturated fatty acids intake, daily zinc intake, vitamin D level, C-reactive protein. OR, odds ratio; CI, Confidence Interval.

**Supplementary Figure 1.** Directed acyclic graph for the association between alcohol consumption and sarcopenia. Exposure: Alcohol consumption. Outcome: Sarcopenia. Minimal sufficient adjustment variables for estimating the total effect of alcohol consumption on sarcopenia: Age, sex, education, ethnicity, married. The references for alcohol consumption-related variables [1-8] and sarcopenia-related variables [9-18] were showed in the supplementary references section. The DAG was constructed using the DAGitty web application at [www.dagitty.net](http://www.dagitty.net) [19].


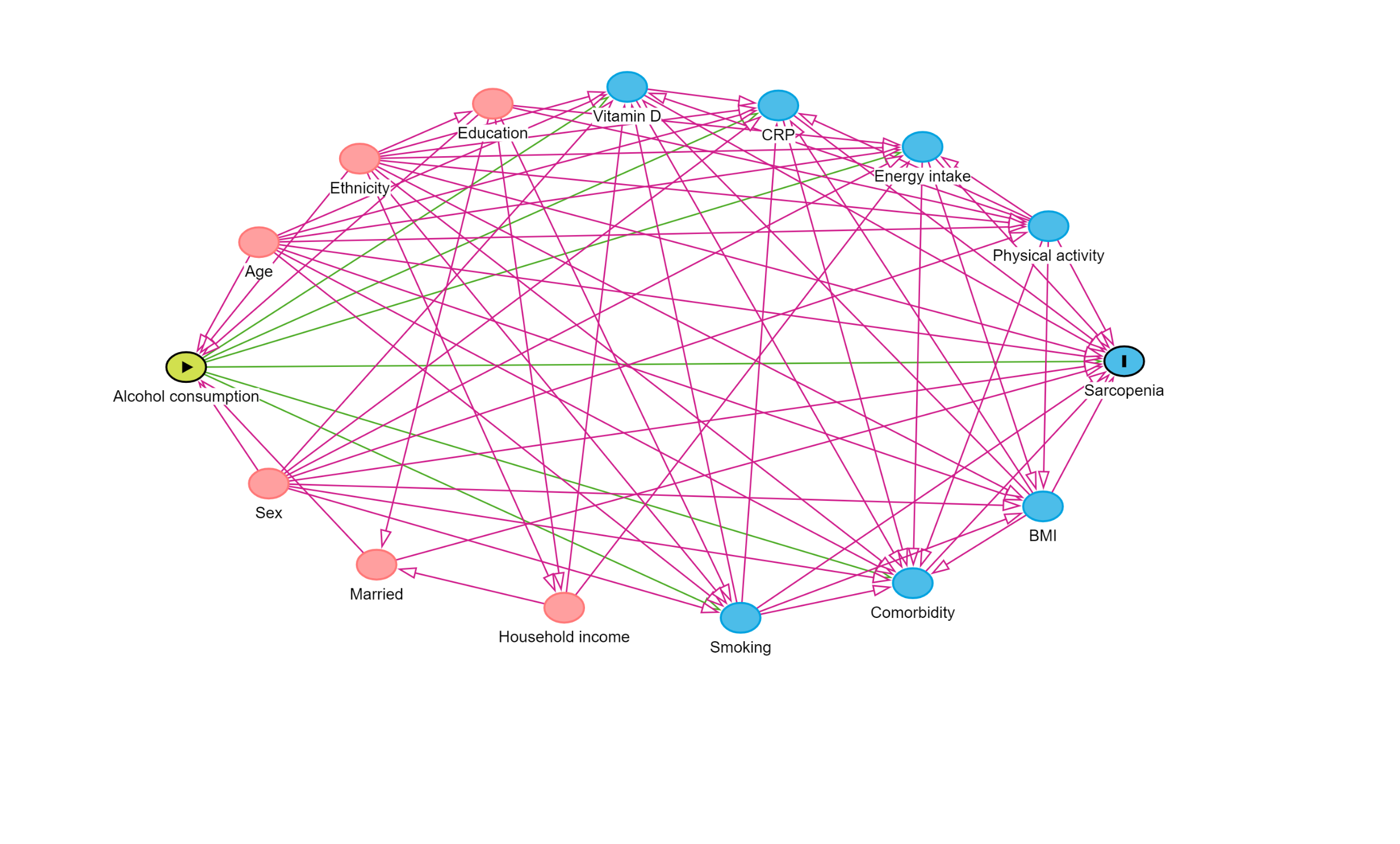


**References**

[1] Naimi TS, Nelson DE, Brewer RD. The intensity of binge alcohol consumption among U.S. adults. Am J Prev Med. 2010;38:201-7.

[2] Carlson CR, Jr., Uriu-Adams JY, Chambers CD, Yevtushok L, Zymak-Zakutnya N, Chan PH, et al. Vitamin D Deficiency in Pregnant Ukrainian Women: Effects of Alcohol Consumption on Vitamin D Status. J Am Coll Nutr. 2017;36:44-56.

[3] Imhof A, Froehlich M, Brenner H, Boeing H, Pepys MB, Koenig W. Effect of alcohol consumption on systemic markers of inflammation. Lancet. 2001;357:763-7.

[4] Yeomans MR. Alcohol, appetite and energy balance: is alcohol intake a risk factor for obesity? Physiol Behav. 2010;100:82-9.

[5] Barbería-Latasa M, Gea A, Martínez-González MA. Alcohol, Drinking Pattern, and Chronic Disease. Nutrients. 2022;14.

[6] Russell AM, Colditz JB, Barry AE, Davis RE, Shields S, Ortega JM, Primack B. Analyzing Twitter Chatter About Tobacco Use Within Intoxication-related Contexts of Alcohol Use: "Can Someone Tell Me Why Nicotine is So Fire When You're Drunk?". Nicotine Tob Res. 2022;24:1193-200.

[7] Pollitt AM, Donnelly R, Mernitz SE, Umberson D. Differences in how spouses influence each other's alcohol use in same- and different-sex marriages: A daily diary study. Soc Sci Med. 2020;264:113398.

[8] Holmila M, Raitasalo K. Gender differences in drinking: why do they still exist? Addiction. 2005;100:1763-9.

[9] Chen H, Ma J, Liu A, Cui Y, Ma X. The association between sarcopenia and fracture in middle-aged and elderly people: A systematic review and meta-analysis of cohort studies. Injury. 2020;51:804-11.

[10] G B, As R. Implications of Race and Ethnicity in Sarcopenia US National Prevalence of Sarcopenia by Muscle Mass, Strength, and Function Indices. Gerontol Geriatr Res. 2021;4.

[11] Remelli F, Vitali A, Zurlo A, Volpato S. Vitamin D Deficiency and Sarcopenia in Older Persons. Nutrients. 2019;11.

[12] Shokri-Mashhadi N, Moradi S, Heidari Z, Saadat S. Association of circulating C-reactive protein and high-sensitivity C-reactive protein with components of sarcopenia: A systematic review and meta-analysis of observational studies. Exp Gerontol. 2021;150:111330.

[13] Park JE, Lee S, Kim K. The effect of combining nutrient intake and physical activity levels on central obesity, sarcopenia, and sarcopenic obesity: a population-based cross-sectional study in South Korea. BMC Geriatr. 2023;23:119.

[14] Liu C, Cheng KY, Tong X, Cheung WH, Chow SK, Law SW, Wong RMY. The role of obesity in sarcopenia and the optimal body composition to prevent against sarcopenia and obesity. Front Endocrinol (Lausanne). 2023;14:1077255.

[15] Lena A, Hadzibegovic S, von Haehling S, Springer J, Coats AJ, Anker MS. Sarcopenia and cachexia in chronic diseases: from mechanisms to treatment. Pol Arch Intern Med. 2021;131.

[16] Locquet M, Bruyère O, Lengelé L, Reginster JY, Beaudart C. Relationship between smoking and the incidence of sarcopenia: The SarcoPhAge cohort. Public Health. 2021;193:101-8.

[17] Hu P, Zhang D, Wong SYS, Woo J, Yu R, Yip BHK, Poon PKM. The Effect of Social Isolation on Sarcopenia: A Longitudinal Study among the Middle-Aged and Older Population in China. Gerontology. 2023;69:748-56.

[18] Dai S, Shu D, Meng F, Chen Y, Wang J, Liu X, et al. Higher Risk of Sarcopenia in Older Adults with Type 2 Diabetes: NHANES 1999-2018. Obes Facts. 2023;16:237-48.

[19] Van Cauwenberg J, De Paepe A, Poppe L. Lost without a cause: time to embrace causal thinking using Directed Acyclic Graphs (DAGs). Int J Behav Nutr Phys Act. 2023;20:145.
